# Age effects on saccadic suppression of luminance and color

**DOI:** 10.1167/jov.21.6.11

**Published:** 2021-06-18

**Authors:** Doris I. Braun, Alexander C. Schütz, Karl R. Gegenfurtner

**Affiliations:** 1 Abteilung Allgemeine Psychologie, Justus-Liebig-Universität Giessen, Giessen, Germany; 2 Abteilung Allgemeine Psychologie, Justus-Liebig-Universität Giessen, Giessen, Germany; 3 Allgemeine und Biologische Psychologie, Philipps-Universität Marburg, Marburg, Germany; 4 Abteilung Allgemeine Psychologie, Justus-Liebig-Universität Giessen, Giessen, Germany; 5 Center for Mind, Brain & Behavior, Marburg, Germany

**Keywords:** visual sensitivity, saccadic suppression, age effects, luminance, color

## Abstract

Saccadic eye movements modulate visual perception: they initiate and terminate high acuity vision at a certain location in space, but before and during their execution visual contrast sensitivity is strongly attenuated for 100 to 200 ms. Transient perisaccadic perceptual distortions are assumed to be an important mechanism to maintain visual stability. Little is known about age effects on saccadic suppression, even though for healthy adults other major age-related changes are well documented, like a decrease of visual contrast sensitivity for intermediate and high spatial frequencies or an increase of saccade latencies. Here, we tested saccadic suppression of luminance and isoluminant chromatic flashes in 100 participants from eight to 78 years. To estimate the effect of saccadic suppression on contrast sensitivity, we used a two-alternative forced choice (2AFC) design and an adaptive staircase procedure to modulate the luminance or chromatic contrast of a flashed detection target during fixation and 15 ms after saccade onset. The target was a single horizontal luminance or chromatic line flashed 2° above or below the fixation or saccade target. Compared to fixation, average perisaccadic contrast sensitivity decreased significantly by 66% for luminance and by 36% for color. A significant correlation was found for the strength of saccadic suppression of luminance and color. However, a small age effect was found only for the strength of saccadic suppression of luminance, which increased from 64% to 70% from young to old age. We conclude that saccadic suppression for luminance and color is present in most participants independent of their age and that mechanisms of suppression stay relatively stable during healthy aging.

## Introduction

During our daily life we hardly think about the consequences of our frequent and mostly unnoticed eye movements, which determine what, when and how we see things or our visual surroundings (for reviews see Kowler, 2011; Schütz, Braun, & Gegenfurtner, 2011; Tatler, Hayhoe, Land, & Ballard, 2011; Gegenfurtner, 2016; Hayhoe, 2017). When we search for something, read a text or look around in our environment, we make sequences of rapid eye movements, so-called saccades, separated by periods of fixation (Yarbus, 1967; Noton & Stark, 1971; Tatler et al., 2011). These discrete eye movements align our line of sight with peripheral objects of interest, so that after each saccade their images (or parts of them) are projected onto a small but highly sensitive area in the center of our retina, the fovea. During each saccadic movement the whole image of the visual scene sweeps across both retinae at high-speed into the direction opposite to the eye movement until the eyes are held again more or less stable during fixation (Martinez-Conde, Macknik, & Hubel, 2000; for reviews see Rolfs, 2009; Rucci & Poletti, 2015). It is fascinating that we hardly ever notice any abrupt visual image displacements or any motion blur between these presaccadic to postsaccadic image transitions (Matin, Clymer, & Martin, 1972; Campbell & Wurtz, 1978; Duyck, Wexler, Castet, & Collins, 2018). Instead, we perceive a clear continuous visual world, in which foci change according to our interests, attention, and actions with seamless transitions (Yarbus, 1967; for reviews see Hayhoe & Ballard, 2005; Land, 2009; Zhao, Gersch, Schnitzer, Dosher, & Kowler, 2012).

How visual stability is achieved is still a matter of debate, but we do know that it is supported by several distinct processes such as peri-saccadic suppression and pre-saccadic remapping (for reviews see Wurtz, 2008; Cavanagh, Hunt, Afraz, & Rolfs, 2010; Rao, Mayo, & Sommer, 2016). Whenever a saccade is planned, our visual system gets prepared already 100–50 ms before saccade onset by shifts of attention to the future target location (Kowler, Anderson, Dosher, & Blaser, 1995; Deubel & Schneider, 1996; Jonikaitis & Belopolsky, 2014), by compression of visual space toward the saccade location (Ross, Morrone, & Burr, 1997; Morrone, Ross, & Burr, 1997; Lappe, Awater, & Krekelberg, 2000; Tolias, Moore, Smirnakis, Tehovnik, Siapas, & Schiller, 2001; Zirnsak, Steinmetz, Noudoost, Xu, & Moore, 2014) and corresponding “remapping” and updating processes (Duhamel, Colby, & Goldberg, 1992; Nakamura & Colby, 2002; Merriam, Genovese, & Colby, 2003; Rolfs, Jonikaitis, Deubel, & Cavanagh, 2011; but see Arkesteijn, Belopolsky, Smeets, & Donk, 2019).

In addition to processes concerning the impending saccade goal there is a marked reduction of visual sensitivity starting up to 100 ms before saccade onset and lasting about 150 to 200 ms (Dodge, 1900; Latour, 1962; Burr, Morrone, & Ross, 1994; Diamond, Ross, & Morrone, 2000; Ibbotson & Krekelberg, 2011; Braun, Schütz, & Gegenfurtner, 2017; Idrees, Baumann, Franke, Münch, & Hafed, 2020; for recent reviews see Morrone, 2014; Binda & Morrone, 2018). The transient attenuation of contrast sensitivity affects the whole visual field, and its strength and duration is determined by the specific condition, i.e. saccade size, test stimuli, illumination and background (Latour, 1962; Zuber & Stark, 1966; Burr, Holt, Johnstone, & Ross, 1982; Diamond et al., 2000; Ross, Morrone, Goldberg, & Burr, 2001; Knöll, Binda, Morrone, & Bremmer, 2011; Schütz, Braun, & Gegenfurtner, 2007; Braun et al., 2017; Idrees et al., 2020). For structured backgrounds perceptual suppression is found for real saccades and saccade-like image shifts (Diamond et al., 2000). The strength of shift effects is modulated by the image statistics and since shift effects are observed in isolated animal retinae, visual sensitivity during saccades is also determined by purely retinal components (Idrees et al., 2020). It is also determined by the combined properties of the visual input to both eyes (Chahine & Krekelberg, 2008).

Some controversy exists with respect to the selectivity of saccadic suppression. Psychophysical studies in humans have reported that during saccades only the contrast sensitivity for flashed luminance gratings of low spatial frequencies (< 0.5 cpd) was reduced by 70–90%, while the sensitivity for gratings with high spatial frequencies and for gratings modulated in chromatic contrast was unaffected during saccades or even enhanced after saccades (Burr, Holt, Johnstone, & Ross, 1982; Morrone, & Ross, 1994; Uchikawa & Sato, 1995; Diamond et al., 2000; Ross et al., 2001; Bruno, Brambati, Perani, & Morrone, 2006; Knöll et al., 2011). In our recent psychophysical study of visual sensitivity during pursuit and saccadic eye movements we found—besides the described saccadic suppression of luminance—also a significant suppression of color sensitivity but no postsaccadic enhancement (Braun et al., 2017). Compared to fixation, contrast sensitivity of low-spatial frequency luminance flashes was reduced up to 90% and for chromatic isoluminant flashes up to 58%, depending on the conditions.

Three primary factors are thought to contribute to saccadic suppression. First, the pre- and post-saccadic images exert visual forward and backward masking and lead to saccadic omission (Matin et al., 1972; Campbell & Wurtz, 1978; Duyck et al., 2018). Second, visual sensitivity is vastly reduced for the high temporal frequency intrasaccadic motion signals (Burr et al., 1982; Castet & Masson, 2000; Castet, 2010). Third, an internal (extraretinal) signal to monitor the oculomotor commands for the initiation of a saccade (Sperry, 1950; von Holst & Mittelstaedt, 1950) initiates the reduction of visual sensitivity even before saccade onset on unstructured backgrounds (Volkmann, 1962; Burr et al., 1994; Diamond et al., 2000). This monitoring signal for the upcoming eye position change by the next saccade is very important for the neural system to disambiguate a self-induced displacement of the retinal image from a displacement caused by an object movement in the external world as suggested by many scientists (for a historical overview see Morrone, 2014). Von Holst and Mittelstaedt (1950) called this internal signal for self-movements “efference copy” (EC), as a copy of the efferent motor command, whereas Sperry (1950) used the term “corollary discharge” (CD) to indicate the concomitant neuronal activity in different neural structures (Sommer & Wurtz, 2006; Wurtz, 2008; Ibbotson & Krekelberg, 2011). Despite the different terms, the theoretical idea of the authors is similar: even before the initiation of an active voluntary movement, the EC/CD is sent to a comparator stage to cancel the incoming retinal motion signals caused by the movement, i.e. the shift of the whole visual field into the opposite direction. More recently two neural circuits, which seem to convey corollary discharge signals have been identified. One circuit includes the frontal eye field and seems to be important for the compensation of saccadic image displacements (Duhamel, Colby, & Goldberg, 1992; Sommer & Wurtz, 2006; Gaymard, Rivaud, & Pierrot-Deseilligny, 1994; Ostendorf, Liebermann, & Ploner, 2010; 2013; Cavanaugh, Berman, Joiner, & Wurtz, 2016). The other circuit includes area MT and seems to contribute to the suppression of visual motion signals during saccades (Robinson & Wurtz, 1976; Cloherty, Mustari, Rosa, & Ibbotson, 2010; Berman, Cavanaugh, McAlonan, & Wurtz, 2017). Spatial-frequency-selective saccadic suppression was also reported for the visual-motor neurons of the superior colliculus (Chen & Hafed, 2017).

So far, little is known about the interplay between vision and eye movements during healthy ageing. There is a massive decline in basic sensory and motor processes during aging. In particular, visual sensitivity is declining with age (e.g., Sekuler & Hutman, 1980; Burton, Owsley, & Sloane, 1993; for reviews see Spear, 1993; Owsley, 2011), and also saccadic parameters such as latency (e.g., Irving et al., 2006; Munoz, Broughton, Goldring, & Armstrong, 1998). Saccades provide continuously high-acuity visual information about selected parts of our surroundings and our body, which is important to control the spatial relationship and position of our body or body parts in relation to each other or objects (Lord, 2006). However, large retinal image shifts caused by saccades have to be suppressed at the right time and long enough to prevent the disturbing perception of blurry moving images when perisaccadic masking effects are not sufficient. For slower image shifts as occurring during pursuit eye movements, visual illusions such as the Filehne illusion (Filehne, 1922; Mack & Herman 1973), the Aubert-Fleischl phenomenon (Aubert, 1886; Fleischl, 1882) or the Duncker illusion (Duncker, 1929) demonstrate that our visual system is not always able to perfectly compensate for self-inducted retinal image shifts. This imbalance between retinal and extraretinal signals seems to increase with age, at least for the Filehne-illusion (Wertheim & Bekkering, 1992; Freeman, Naji, & Margrain, 2002). An inability to suppress moving blurry images around saccades may induce a sense of self-motion and induce body sway.

Our aim was to investigate whether and how mechanisms of saccadic suppression would be affected by healthy ageing and whether and to what extent saccadic suppression for color would be present in a larger population. Age-related effects on saccadic suppression of contrast sensitivity were studied so far only by Bruno, Brambati, Perani and Morrone (2006) in children and adolescents (8–18) and young adults (18–31). These authors found that saccades reduce perisaccadic contrast sensitivity for luminance by 70% to 90% for adults and by 90% to 95% for children and adolescents. The authors suggested that the stronger saccadic suppression found in children might reflect the immaturity of their oculomotor system. They did not find significant saccadic suppression for color at the group level, but a third of their observers did show suppression for color of around 30% to 40%. Here, we measured contrast sensitivity for luminance and color during fixation and shortly after saccade onset in a large group of naïve participants of different ages.

## Methods

### Participants

**Figure 1.**
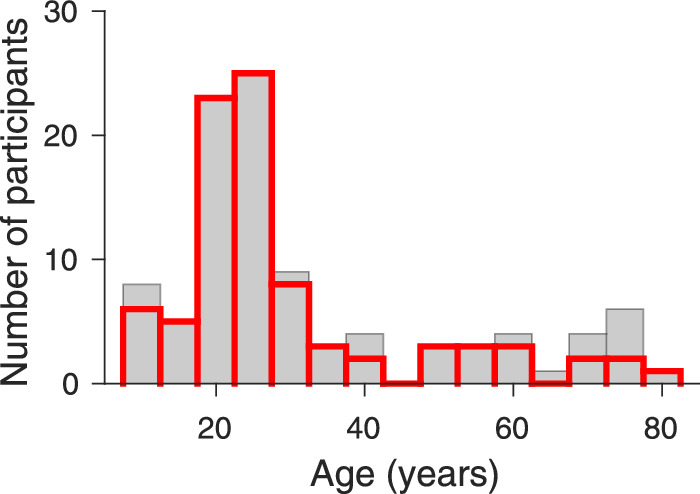
Age histogram of the participants included in the data analysis of the luminance experiment (N = 99; average age, 32.35 years, gray bars), and of the color experiment (N = 86; average age, 29.22 years, red open bars). For 85 participants we were able to collect data of both experiments.

One hundred nine untrained naïve participants were recruited by ads in the university network or by handouts or private contacts. Many participants were students or employees of Giessen University, and some of the older subjects were recruited from the database of a large age study project at Giessen University (e.g., Huang, Gegenfurtner, Schütz, & Billino, 2017; Valsecchi, Billino, & Gegenfurtner, 2018). Adolescents were mostly children of colleagues. All participants had normal or corrected to normal vision and no major health issues. They were naïve regarding the purpose of the experiments. After a short general introduction with respect to eye movements, the task and equipment, we tested participants for color vision deficiencies with Ishihara's tests for color deficiencies, the 24-plates edition (Ishihara, 2018) and conducted a short interview about the general health, age, education, and profession. Four of the 109 participants did not finish any of the testing because they had either problems sitting for a longer time in a fixed position or could not keep their eyes stable on the fixation point because of larger drifts or sleepiness or because their glasses were reflecting too much. Three of these excluded participants were older than 60 years. One was 12 years old and too tired for the test after school. For the luminance experiment we excluded five participants because we could not fit psychometric functions to their data. During the testing of one participant a technical problem occurred during the experiment with luminance stimuli and we could only analyze the data of the experiment with color stimuli. Thus we successfully tested and analyzed data for 99 participants with the luminance stimuli. Their average age was 32.35 (median, 25; range, 8–78 years); 42 of them were male (Figure 1). Forty-three participants were tested with a neutral gray and 56 with a gray-greenish screen background. Three participants had a red-green color deficiency and were therefore only tested in the luminance experiment.

We could test 97 of the participants (without color deficiencies) also with the color stimuli and excluded 11 participants because no psychometric functions could be fitted to their data. The excluded 11 participants were between 38 and 75 years old (average, 62.91 years). For the remaining 86 participants (average age, 29.22 years; median, 24; SD 15.94; 31 males) chromatic color sensitivity was successfully tested and analyzed (Figure 1). Twenty-nine participants were tested with a neutral gray background and 57 with the gray-greenish background. Experiments were approved by the local ethics committees (Giessen LEK 2013-0018, Marburg 2015-35k) and were in line with the declaration of Helsinki. All participants and their legal guardians in the case of the children signed an informed consent form at the beginning of the experiment. They were paid for participation and received information about their results after the experiments, i.e. the measured contrast sensitivity for luminance and color and the amount of suppression.

### Procedure

**Figure 2.**
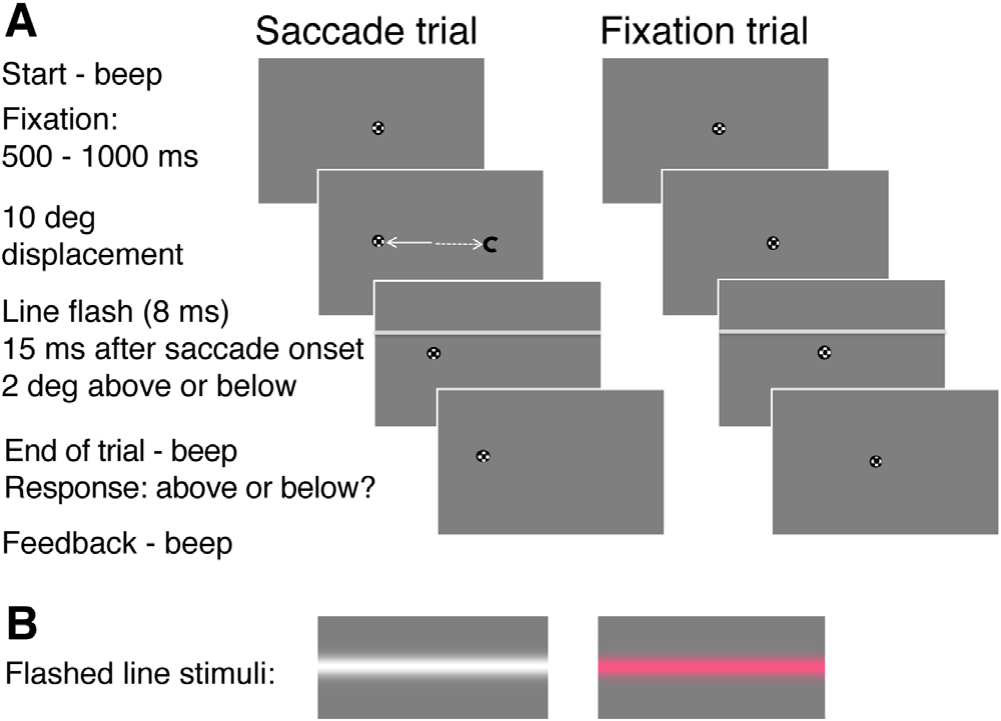
Experimental conditions and time sequence of the tasks. Detection rates for the position of luminance or isoluminant red line flashes were measured either during horizontal saccades or central fixation. Each trial started with a beep and the appearance of a fixation target for 500 to 1000 ms in the screen center. In a saccade trial the fixation target was displaced 10° to the left or right; in a fixation trial it remained in place; both trial types were randomly intermixed. Participants had the task to keep their eyes on the fixation/saccade target. A horizontal line was flashed for 8 ms across the whole screen 2° above or below the screen center 15 ms after saccade onset or 500 to 1000 ms after fixation trial onset. A beep indicated the end of each trial and participants indicated or guessed the line position by pressing one of two assigned keys on a keyboard.

Participants viewed the stimuli on the monitor binocularly with their heads stabilized by an adjustable chin- and forehead-rest. Both experiments were conducted in a dark room illuminated only by the monitors for the experimental display and the eye movement supervision. Before each experiment, each participant received a short introduction about the task and time sequence of the stimuli by explaining an illustration of the experiment on a printout. After final adjustments of chair and head position, the task and the function of the computer keys were clarified again with five example trials shown on the monitor screen. Then an eye tracker calibration was performed for each participant. Participants initiated each trial by pressing the space bar. They were instructed to keep their eyes always on the single black target; either to fixate it when it stayed in the center of the screen (fixation condition) or to follow and to re-fixate it after a sudden horizontal step of 10° unpredictably to the left or right (saccade condition). The saccade target location was randomized to avoid anticipatory saccades which are frequent when the target location is kept constant. This also facilitates the comparison of our data with other studies about age effects on saccades (Munoz et al., 1998). The horizontal line stimulus was flashed 2° above or below the fixation/saccade target across the whole screen 500 to 1000 ms after trial onset during central fixation or 15 ms after saccade onset (see Figure 2). Because saccadic mislocalization orthogonal to the saccade direction is much smaller than along the saccade direction (Kaiser & Lappe, 2004), and because the vertical distance of detection stimulus to the saccade target was 2°, we did not expect mislocalization to affect our task. A low beep indicated the end of each trial and participants were asked to indicate whether they had seen the flashed line above or below the fixation/saccade target by pressing one of two response keys. Feedback for a wrong answer was provided by a low-pitch beep. An adaptive staircase procedure with a one-up/two-down rule (Levitt, 1971) adjusted luminance or chromatic contrast of the line stimulus according to the participant's response separately for the fixation and saccade condition. To keep the testing time as short as possible for the children and senior adults, we limited the number of trials for each experiment to 160 (80 trials for fixation and 80 trials for saccades). Because participants initiated each trial, they could control the timing between trials and stop whenever they wanted. In most cases a single experiment lasted between 20 to 25 minutes, and the two experiments were typically completed in less than one hour with a longer break in between. Children decided whether they wanted to do one or two experiments on the same day. The sequence of the two experiments was randomized.

Because our aim was to investigate the presence of saccadic suppression of luminance and color in a large group of participants of different ages, we tested naïve, untrained participants. We chose a very basic saccade task, in which the saccade target appeared randomly left or right from central fixation. Making visually guided saccades to isolated and well-defined visual targets, which suddenly appear, is a very simple task and therefore often used in age studies without any training sessions. The randomization of the target location has the advantage that anticipatory saccades occur less frequent. This was important for us, because we wanted to measure the latencies of visually guided saccades only to test for age effects and we needed as many valid saccades as possible for the psychometric functions during a one-hour test session.

### Apparatus

Stimuli were presented on a Display++ LCD monitor (Cambridge Research Systems Ltd., Riverside, Kent, UK) driven at a 120-Hz refresh rate. At a viewing distance of 90 cm the active screen area subtended 42.5° horizontally and 24.45° vertically on the participant's retina and with the spatial resolution of 1.920 × 1.080 pixels this results in 45 pixels/deg. For the control of the stimulus presentation we used the Psychtoolbox (Brainard, 1997; Pelli, 1997; Kleiner, Brainard, & Pelli, 2007). In the first part of the study we used for both experiments a neutral gray background (neutral gray: 74.14 cd/m^2^, x = 0.2863, y = 0.3067) and completed the experimental testing with 43 participants. Because in the color experiment 11 of the tested participants had more problems to perceive the isoluminant red line flash during saccades, we decided to increase the chromatic contrast range. We changed the gray background color for both experiments to a slightly darker more greenish-gray (55.73 cd/m^2^, x = 0.2474, y = 0.3230) and tested 57 participants with a gray-greenish background. Color calibrations and measurements of the monitors' gamma curves were carried out with a Konica Minolta Spectroradiometer CS-2000A (Konica Minolta Holdings Inc., Marunouchi, Tokyo, Japan). Stimuli were gamma corrected before they were displayed on the monitor.

### Stimuli

Black circular stimuli were used as targets for fixation and saccades, consisting of a bull's eye combined with a cross-hair (Thaler, Schütz, Goodale, & Gegenfurtner, 2013) with an outer circle diameter of 0.6° and an inner circle of 0.2°. To measure contrast sensitivity during fixation and saccades we used a horizontal line stimulus to provide a similar retinal image during the fixation and saccade condition. Our single line stimulus was specified vertically by a Gaussian distribution with a standard deviation of 0.15°. The spatial frequency profile of the line is therefore also Gaussian with a standard deviation of 0.15/2π = 1.06 cpd so that most of its energy was clearly below 1 cpd (see Schütz, Braun, Kerzel, & Gegenfurtner, 2008). This low-spatial frequency line was flashed for one refresh cycle of the monitor (8 ms) either 2° above or below the fixation/saccade target across the whole monitor screen. Although this particular stimulus does not lead to maximal saccadic suppression, it does avoid floor and ceiling effects. Therefore, it was suited to study changes of saccadic suppression with age, which could go in both directions. The contrast of the luminance line stimulus was modulated along the L+M axis; the chromatic contrast of the isoluminant red line was modulated along the L-M axis of the DKL color space and defined in cone contrast space (Derrington, Krauskopf, & Lennie, 1984, Gegenfurtner & Hawken, 1996). Isoluminance was defined by the V(λ) curve and verified with the spectroradiometer. Because at photopic light levels visual sensitivity for stimuli of low spatial frequency is not or only minimally impacted by healthy aging (Owsley, 2011) we expected only a modest increase of contrast thresholds during the fixation condition with age.

### Eye movement recording and data analysis

**Figure 3.**
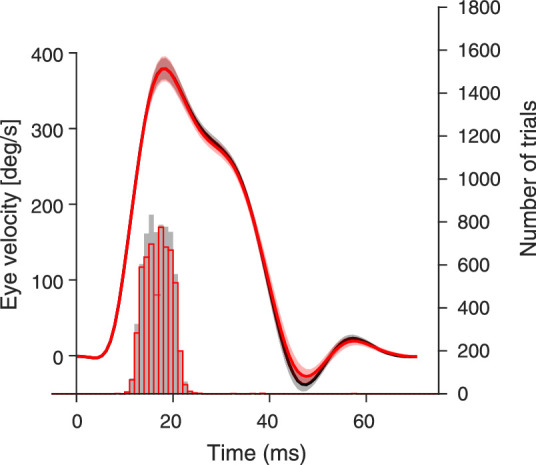
Histograms of detection line onsets relative to saccade onsets (right y-axis) and the averaged eye velocities of saccades (left y-axis). For saccade trials, the timing of the 8 ms presentation of the detection stimulus, the horizontal luminance or chromatic line was set to 15 ms after the detected saccade onsets. The histograms show the onset times of the flashed line stimuli for all participants in the luminance (gray bars) or color (red bars) experiment. For the average eye velocity traces the average position traces of each participant were used to calculate the eye velocities for the luminance (gray line) and the color (red line) experiment separately. The shaded areas represent the 95% confidence interval across participants.

The eye position signals of each subject were recorded at 1000 Hz with the Eyelink 1000 Desktop Mount (EyeLink 1000; SR Research Ltd., Osgoode, Ontario, Canada), a video-based infrared eye tracker. The eye tracker was controlled with the Eyelink Toolbox (Cornelissen, Peters, & Palmer, 2002). For the timing of the detection line stimulus, saccade onsets were detected online, when two consecutive velocity samples exceeded 50°/s and 100°/s, respectively. After each experimental session eye position signals were filtered and saccades were detected with the EyeLink saccade detection algorithm and differentiated over time to obtain the eye velocity signals. For each participant, we checked that the stimulus was always flashed during an interval of high eye speed. As shown in Figure 3, the flashes of detection lines occurred well after saccade onsets and coincided most often with the time when saccades reached the peak eye velocity.

Trials were excluded from further analysis based on the following criteria. We excluded trials in the fixation condition, if a saccade or a blink occurred in a time window of ± 100 ms around the line flash. In the saccade condition a trial was excluded if a saccade was not detected, if the saccadic amplitude was smaller than 7.5° or larger than 15°, or its latency was below 100 or above 500 ms or if the eye speed during stimulus presentation was below 50°/s or larger than 500°/s. For each participant detection contrast sensitivity for luminance and color was determined for the fixation and the saccade condition by fitting psychometric functions to the proportion of correct answers with respect the location of the flashed parafoveal line. We used the psignifit toolbox for Matlab (Wichmann & Hill, 2001; Schütt, Harmeling, Macke, & Wichmann, 2016). Contrast sensitivity was defined as the inverse of the threshold value for contrast. The magnitude of saccadic suppression was calculated as 1 − (CS_sacc_/CS_fix_), where CS_sacc_ is the contrast sensitivity measured during the saccade and CS_fix_ is the contrast sensitivity measured during fixation.

## Results

**Figure 4.**
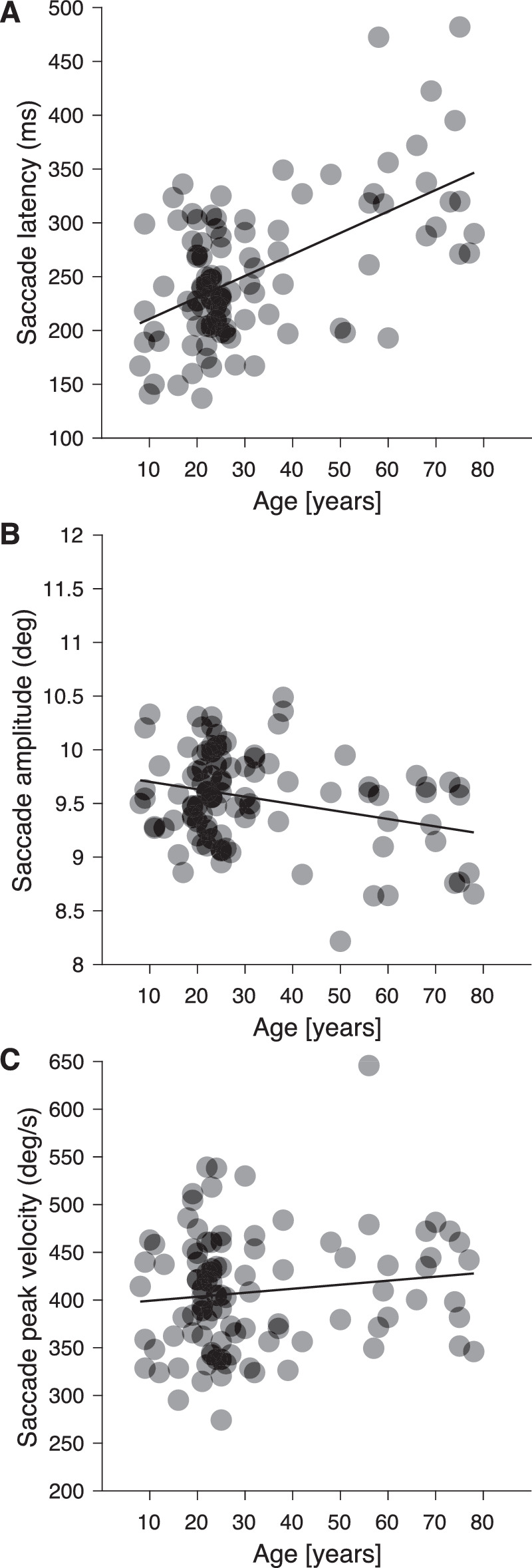
Metrics of all horizontal saccades of 99 participants measured in the luminance experiment plotted with respect to their age. In each graph the black line represents the linear regression plotted to the data. (A) Median of saccadic latencies. (B) Median of amplitudes for horizontal saccades to the targets at 10° eccentricity. (C) Median of the peak eye velocities.

Our aim was to compare contrast sensitivity during fixation and saccades for luminance and color for a large group of naïve participants and to investigate age-related effects on the strength of saccadic suppression. Before going into the perceptual results, we will consider effects of aging on motor aspects of the saccades. In the graphs of Figure 4 three metrics of all horizontal saccades to the saccade targets appearing unpredictably on the left or right at 10° are plotted with respect to the age of the participants for the luminance experiment: the medians of the saccade latency, the saccade amplitudes and the peak eye speeds. Figure 4A replicates the well-known gradual increase of saccades latencies with age (Spooner, Sakala, & Baloh, 1980; Abel, Troost, & Dell'Osso, 1983; Munoz et al. 1998; Peltsch, Hemraj, Garcia, & Munoz, 2011). The correlation of the median latencies with age was significant (ρ_97_ = 0.57, *p* < 0.001). To test further for age effects, we determined two age groups and compared their data: young adults below 40 years (≥18 < 40 years) and senior adults older than 40 years. A split at 40 years of age seems reasonable because changes in contrast sensitivity for intermediate to high spatial frequencies are reported mainly for adults older than 40 years (Elliott, Whitaker & MacVeigh, 1990; Owsley, 2011). Age-related changes are also known for saccade parameters, here a gradual and continuous shift to longer saccadic latencies and durations and a decrease of express saccades is noticeable for subjects older than 40 years (Munoz et al., 1998). For the luminance experiment, the median saccadic latencies in the two age groups, the 63 young adults and the 23 senior adults were 235 ms and 319 ms and the distribution differed significantly (Mann-Whitney U-test, z = −4.7, *p* < 0.0001). The medians of saccadic amplitudes (Figure 4B) decreased significantly with age, for young adults with median amplitude was 9.68° and for senior adults 9.33° (Mann-Whitney U-test, *z* = 3.26, *p* < 0.001). The median saccade peak velocity was 408.2°/s for young adults and 435.1°/s for senior adults but the difference was not significant (Mann-Whitney U-test, *z*  = −1.13, *p* > 0.1).

**Figure 5.**
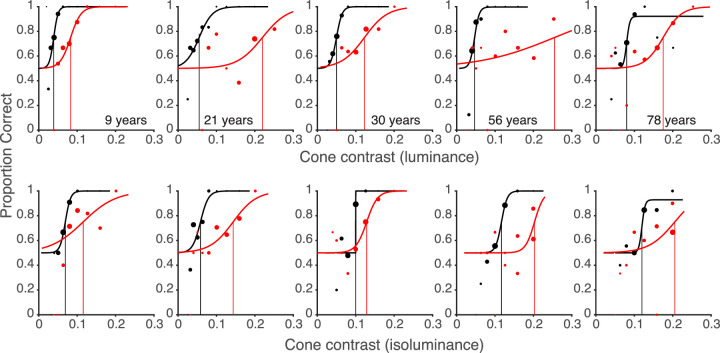
Dependence of the proportions of correct responses with respect to the location of luminance (top) or chromatic (bottom) line flashes during fixation (black curve) and saccades (red curve) on cone contrast for 5 typical participants of different ages. The rightward shifts of the psychometric functions for correct detections during saccades compared to the functions for detection during fixation indicate the amounts of saccadic suppression for luminance (top) or color (bottom). For the five participants suppression for luminance was larger than suppression of color (from left to right, 53% vs. 41%, 73% vs. 60%, 57% vs. 25%, 82% vs. 45%, 55% vs. 41%).

In Figure 5 we show examples of psychometric functions for five participants of different ages for the detection of parafoveal line target flashes during fixation and saccades. The examples illustrate the main findings we will report in detail in subsequent sections: (1) Contrast thresholds are increased dramatically during saccades (red curves) compared to fixation (black curves). (2) This suppression is stronger for luminance (top row) than for color stimuli (bottom row). (3) Suppression is variable between different observers but is little affected by age.

We will first present the results for luminance stimuli, then for color stimuli and then investigate the relationship between the two.

### Saccadic suppression of luminance flashes

**Figure 6.**
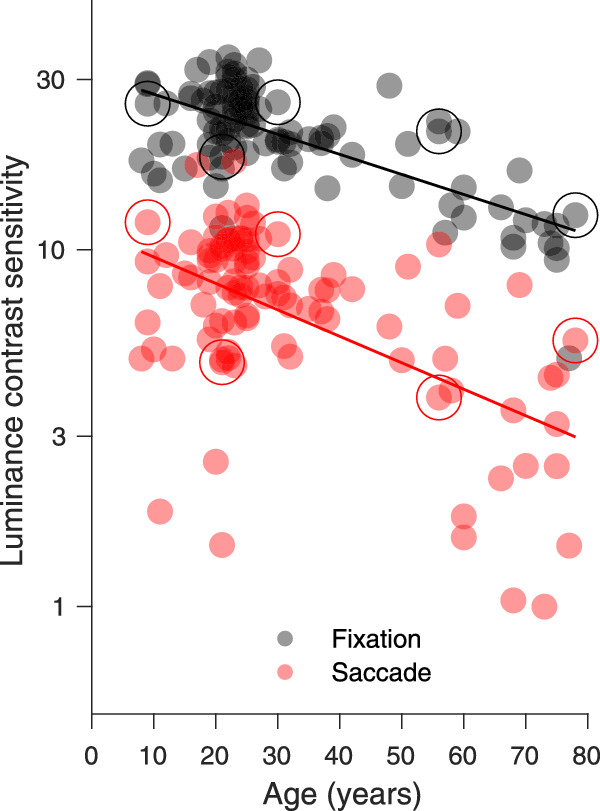
Contrast sensitivity for the detection of parafoveal luminance flashes during fixation and saccades as a function of age (N = 99). The data of five participants, whose psychometric functions are shown in Figure 5, are marked by large circles.

In Figure 6, contrast sensitivity during fixation and saccades is plotted as a function of age for the 99 participants. During fixation, contrast sensitivity for parafoveal luminance flashes was on average 21.54 (standard deviation [SD], 6.45) and during saccades 7.44 (SD, 3.29); that is, during saccades detection contrast sensitivity for luminance decreased by 65% on average (t_98_ = 28.07; *p* < 0.0001). This is in line with numerous previous experiments on saccadic suppression for these kinds of targets (e.g., Bruno et al., 2006; Braun et al., 2017). It has also been shown before that luminance contrast sensitivity during fixation (gray points) decreases with age (Owsley et al., 1983; for review see Owsley, 2011). In our case, the correlation between age and log contrast sensitivity during fixation was ρ_96_ = −0.71 (*p* < 0.0001). A similar negative correlation with age was also present for log luminance contrast sensitivity^1^ during saccades (ρ_96_ = −0.56, *p* < 0.0001). The comparison of the luminance contrast sensitivity of 63 young adults (≥18 < 40 years; average age, 25.11 years; SD, 5.35) with 23 senior adults (≥40 years; mean age, 63.7 years; SD, 10.4) revealed significant differences for contrast sensitivity measured during fixation (young: mean 23.76, SD 4.99 median 23.11, interquartile range [IQR], 7.22; old: mean 14.49, SD, 5.44, median 12.5, IQR 7.12, Mann-Whitney *z* = 5.44, *p* < 0.0001) and for contrast sensitivity measured during saccades (young: mean 8.39, SD 2.71, median 8.12, IQR 3.61, old: mean 4.40, SD 2.63, median 4.03, IQR 3.63; Mann-Whitney *z* = 5.06, *p* < 0.0001). For the 13 adolescents between 8 and 17 years (average age, 12 years; SD, 3.11) the median of luminance detection contrast sensitivity during fixation was 25.66 (IQR 10.22) and during saccades 8.36 (IQR 4.69); therefore during saccades their contrast sensitivity was reduced by a factor of 3.07.

**Figure 7.**
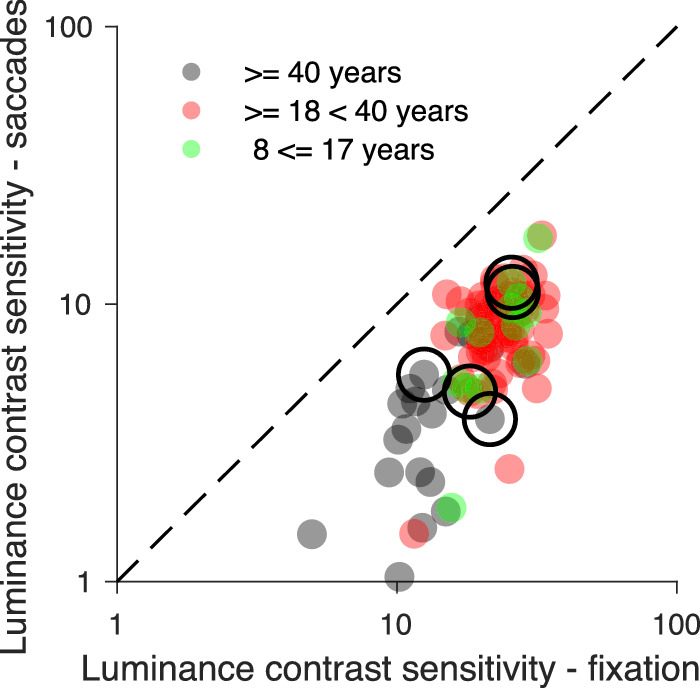
Comparison of contrast sensitivity for luminance flashes during fixation and saccades for 99 participants. Contrast sensitivity data of 23 senior adults (average age, 63.7 years) are plotted in black; of 63 young adults (average age, 25.11 years) in red, and of 13 adolescents (average age, 12 years) in green. The data of five participants, whose psychometric functions are shown in Figure 5, are marked by large circles.

In the scatterplot of Figure 7 luminance contrast sensitivity during fixation is compared to the contrast sensitivity during saccades for all participants and data are colored differently for the three age groups. All data lie below the identity line, indicating that luminance contrast sensitivity of each participant was reduced during saccades compared to fixation. Luminance contrast sensitivity of most senior participants was lower during fixation and saccades compared to young participants. Although the distributions of the contrast sensitivity data of young (red dots) and senior (black dots) adults overlap to a modest extent, the average contrast sensitivity of the young adults is higher for both conditions. The distribution of the luminance contrast sensitivity data for the 13 adolescents was closer to that of the young adults (≥18 and <40 years) but it also overlapped with the distribution of senior adults. Figure 7 also illustrates the correlation between contrast sensitivity during fixation and during saccades (ρ_96_ = 0.65, *p* < 0.001).

**Figure 8.**
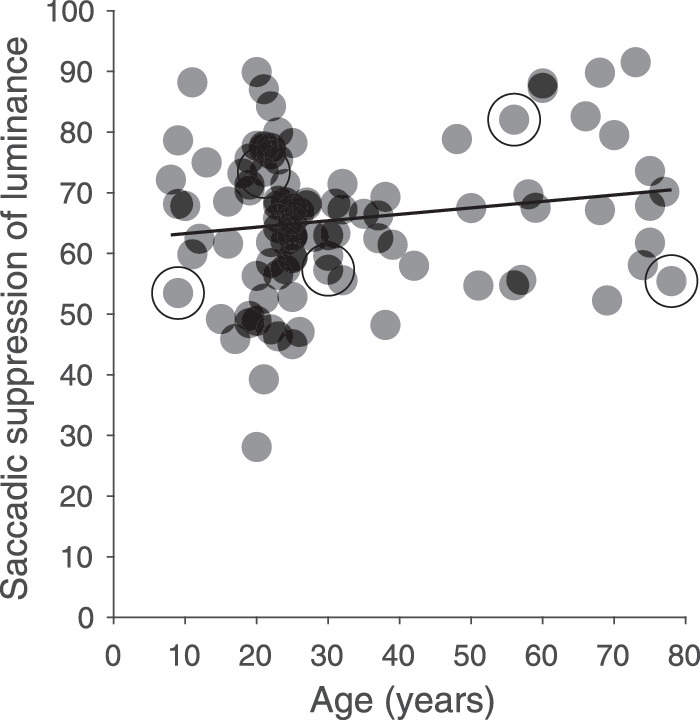
Saccadic suppression of luminance as a function of age (N = 99). The data of five participants, whose psychometric functions are shown in Figure 5, are marked by large circles.

Figure 8 shows that the strength of saccadic suppression of luminance sensitivity only had a modest, nonsignificant correlation with age (ρ_97_ = 0.17, *p* = 0.1). The average magnitude of saccadic suppression for luminance was 65.64 % (SD 12.1; median, 66.82; IQR, 15.83). The comparison of saccadic suppression between young adults (mean 64.03%; SD, 11.7; median, 65.55%; IQR, 14.09) and senior adults (mean 70.17%, SD 12.69; median 67.85; IQR, 23.42) revealed a small but significant difference (t_84_ = 2.11, *p* = 0.038). The strength of saccadic suppression of the 13 adolescents showed strong individual differences but on average their luminance suppression with a mean of 65.47% (SD 11.89) was similar to the average suppression of young adults. For the nine children and young adolescents younger than 15 years, there was a trend for slightly higher suppression (69.53%). Figure 8 illustrates nicely that all the individual data points for the older observers fell well within the range of values observed in the younger group.

### Saccadic suppression of isoluminant red flashes

**Figure 9.**
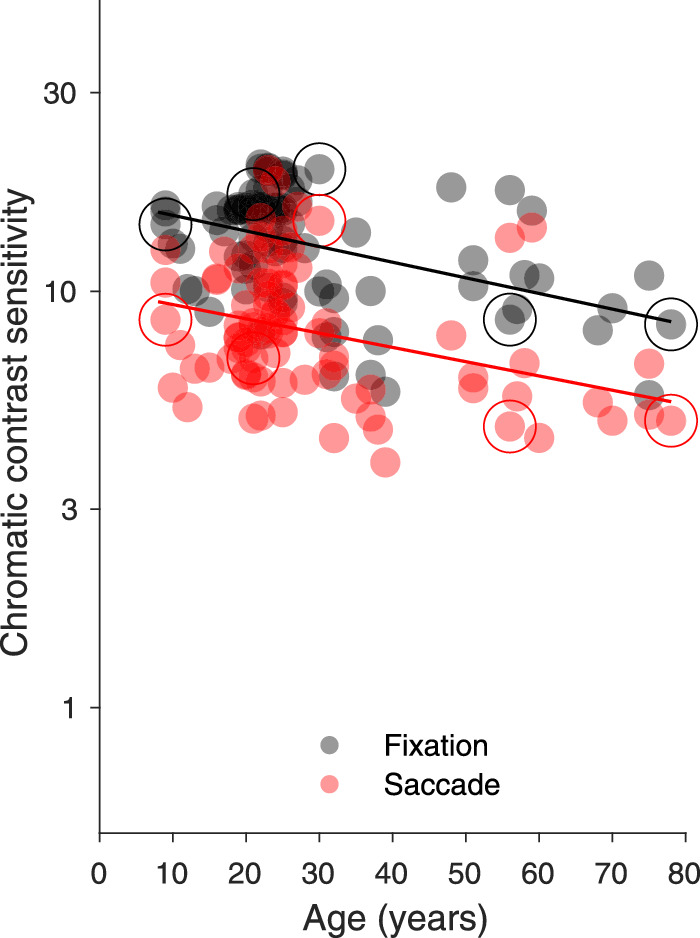
Chromatic contrast sensitivity for the detection of parafoveal isoluminant red flashes during fixation (black circles) and saccades (red circles) as a function of age (N = 86). The data of five participants, whose psychometric functions are shown in Figure 5 are marked by large circles.

Chromatic contrast thresholds for the detection of parafoveal isoluminant flashes were measured during fixation and saccades for 86 participants (see Figure 9). During fixation the average chromatic contrast sensitivity (expressed in cone contrast units) for the isoluminant red flashes was 13.50 (SD 3.84; median 14.08; IQR, 5.81) and decreased during saccades to 8.54 (SD 3.30; median 7.88; IQR, 4.36). The difference between the chromatic contrast sensitivity measured during fixation and saccades was highly significant (Mann-Whitney *z* = 7.96, *p* < 0.0001). As described in the literature (Knoblauch, Vital-Durand, & Barbur, 2001; Paramei & Oakley, 2014; Barbur & Rodriguez-Carmona, 2016) a decrease of chromatic contrast sensitivity with increasing age was present for the fixation condition (ρ_84_ = −0.44, *p* < 0.001). Our data show also a decrease of chromatic contrast sensitivity for the saccades condition (ρ_84_ = −0.35, *p* < 0.001). During fixation the average chromatic contrast sensitivity for the 61 young adults was 14.11 (SD 3.90; median 15.59; IQR, 5.16) and for the 14 senior adults 11.03 (SD 3.60; median 10.52; IQR, 3.32). During saccades chromatic contrast sensitivity for young adults decreased to 8.87 (SD 3.37; median 8.01; IQR, 4.13) and for senior adults to 6.86 (SD 3.10; median 5.72; IQR, 1.83). The comparison of the chromatic contrast sensitivity of young versus senior adults revealed a significant difference for measurements during fixation (Mann-Whitney *z* = 2.52; *p* < 0.01; degree of freedom, 73) and during saccades (Mann-Whitney *z* = 2.77; *p* < 0.01). For the 11 adolescents the mean chromatic contrast sensitivity during fixation was 13.24 (SD 2.54; median 13.94; IQR, 4.62) and during saccades 8.83 (SD 2.66; median 8.55; IQR, 4.18). When comparing the decline in sensitivity during fixation for luminance and for color (see Figures 9 and 4), the decline is shallower with age for the chromatic stimuli. Sensitivity to luminance decreases from 23.77 for the young adults to 14.50 for the older participants. For color, the decrease is from 14.11 to 11.03, which is much more modest. This result is in line with recent data by Mateus, Lemos, Silva, Reis, Fonseca, Oliveiros, and Castelo-Branco (2013), but it is contrasting with evidence that the retinogeniculate parvocellular system is more affected by aging than the magnocellular system (Elliott & Werner, 2010).

**Figure 10.**
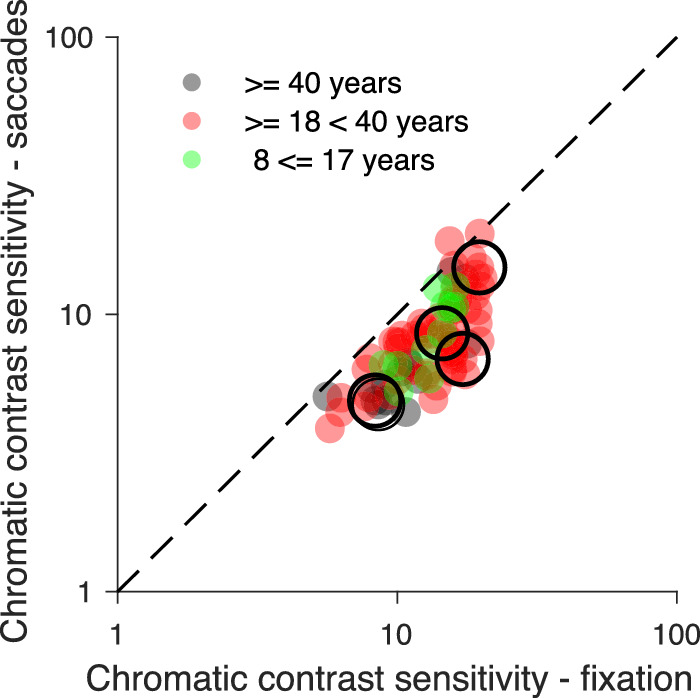
Comparison of chromatic contrast sensitivity during fixation and saccades of 86 participants. Chromatic sensitivity data of the 14 senior adults are plotted in black, of the 61 young adults in red and of 11 adolescents in green. The data of the five participants, whose psychometric functions are shown in Figure 5, are marked by large circles.

In the scatterplot of Figure 10 chromatic contrast sensitivity during fixation is plotted relative to the chromatic sensitivity during saccades for each participant and colored differently for the three age groups, as in the scatterplot for luminance contrast sensitivity shown in Figure 7. Compared to the scatterplot for luminance sensitivity most data for chromatic contrast lie below but closer to the identity line, indicating that the reduction of chromatic contrast sensitivity during saccades is smaller. The chromatic contrast sensitivity data of the 14 senior adults (≥40 years), the 61 young adults (≥18< 40 years), and the 11 adolescents below 18 years largely overlap.

**Figure 11.**
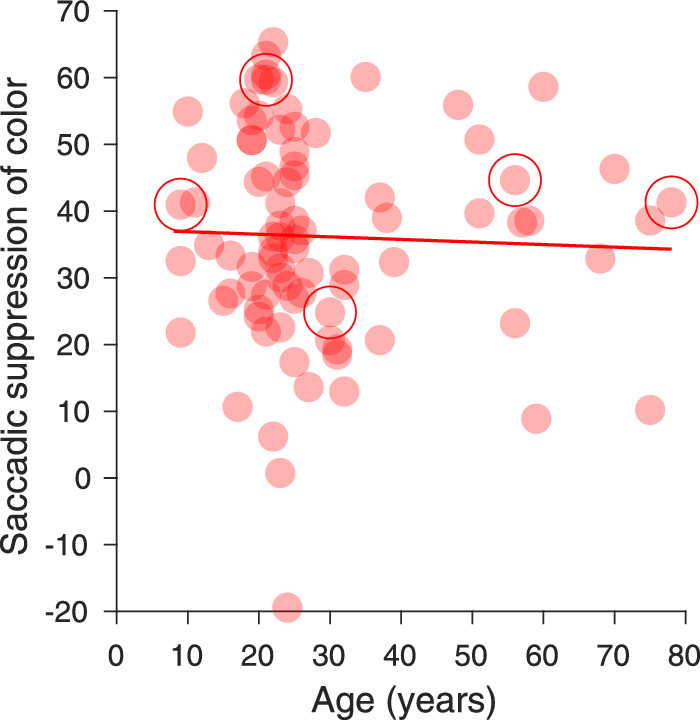
Saccadic suppression of chromatic contrast sensitivity as a function of age (N = 86). The data of the five participants, whose psychometric functions are shown in Figure 5, are marked by large circles.

Overall, saccadic suppression of color contrast thresholds was 36.16% (SD 15.79; median 36.12; IQR, 21.09) and therefore smaller than the 65.64% saccadic suppression for luminance (SD 12.11; median 66.82; IQR, 15.83). It also showed more intersubject variability, as seen in Figure 11. One participant could detect color flashes slightly better during saccades, and another participant showed no difference between fixation and saccades. Only one participant had a color suppression larger than 65.64%, the average value for luminance suppression. No age effect was found for the strength of chromatic suppression (ρ_84_ = −0.04, *p* = 0.72). Young adults had an average suppression of chromatic sensitivity of 36.22% (SD 16.67; median 35.79; IQR, 24.16), senior adults an average suppression of sensitivity of 37.69% (SD 14.94; median 39.12; IQR, 13.43) and the 11 adolescents an average suppression of 33.86% (SD 12.35; median 33.33; IQR, 14.38), the differences among the three groups were not significant.

### Comparison of saccadic suppression of luminance and color

**Figure 12.**
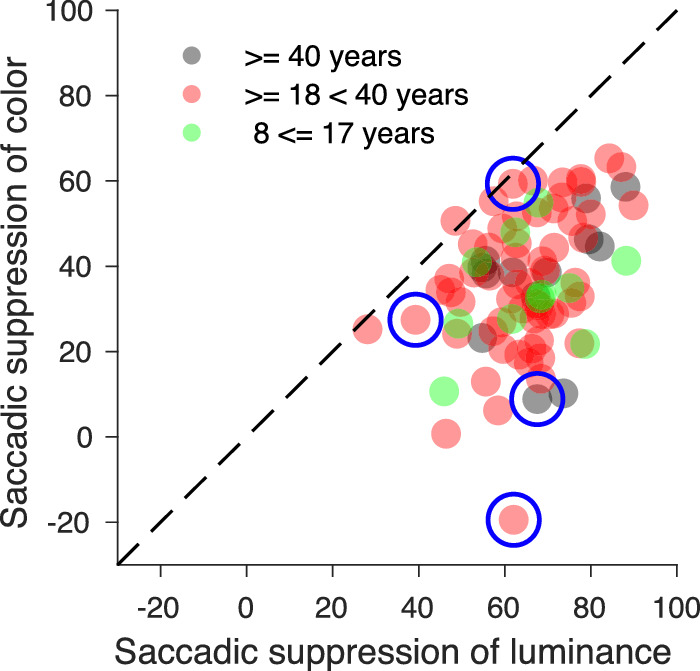
Comparison of saccadic suppression for luminance and color for participants (N = 85) who finished both experiments. Data of 61 young adults are plotted in red, data of 13 senior adults in black and those of 11 adolescents in green. The suppression data of the four participants, whose psychometric functions are shown in Figure 13 are marked by large blue circles.

For 85 participants we were able to measure saccadic suppression for both luminance and color stimuli. The comparison of the saccadic suppression of luminance and color for the same participants revealed a highly significant correlation (ρ_83_ = 0.37; *p* < 0.001). The scatterplot of Figure 12 also shows that average luminance suppression (mean 65.64%; SD 12.11; median 66.82; IQR, 15.83) was stronger than average color suppression (mean 36.16%; SD 15.79; median 36.12; IQR, 21.09). This held for nearly all participants; i.e. all but one of the data points lie below the dashed unity line. The difference was highly significant (t_84_ = 16.9; *p* < 0.0001). Age does not seem to have a strong influence since the distributions of the three age groups overlapped to a large extent. Individual differences with respect to the strength of saccadic suppression were more pronounced for color than for luminance stimuli.

**Figure 13.**
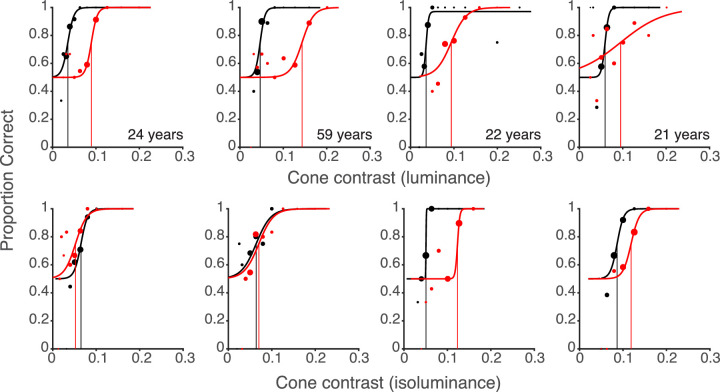
Psychometric functions of four participants for the detection contrast sensitivity for luminance (top) and for color flashes (bottom) during fixation (black curve) and during saccades (red curve). The participants have different saccadic suppression for color (from −19% to 63%) but high suppression for luminance (from 62% to 87%). The data of the four participants are marked by blue circles in Figure 12.

To illustrate the variability of chromatic suppression, psychometric functions of 4 participants are presented in Figure 13. The first participant could see color slightly better during saccades by 19%, the second one had only little suppression (8%), the third and the fourth participant had similar strong color suppression (58.82% and 63.86%) similar or slightly less than their luminance suppression.

## Discussion

We measured contrast sensitivity for luminance and color during fixation and saccades of 100 naïve participants from eight to 79 years of age to investigate the general presence of saccadic suppression for both and to investigate the effects of healthy aging. The comparison of contrast sensitivity for parafoveal luminance or isoluminant chromatic flashes during fixation and 15 ms after the onset of horizontal 10° saccades revealed a significant suppression of 66% for luminance and a smaller but significant suppression of 36% for color. Although luminance and chromatic contrast sensitivity during fixation and saccades decreased significantly with age, the strength of saccadic suppression was rather stable and increased slightly with age for luminance only. Adolescents showed larger individual differences, but on average, their saccadic suppression for luminance and color was similar to that of young adults. Overall, we found that with few exceptions saccadic suppression is present for both, luminance and color in a large group of participants, that strong individual differences with respect to the strength of saccadic suppression are present and that a significant correlation exists between the strength of suppression for luminance and color across observers. Mechanisms of saccadic suppression show considerable variability during adolescence, which might indicate fine-tuning and maturation of the visual and oculomotor system, however, during healthy aging they remain relatively stable.

### Age effects of saccadic suppression

Effects of healthy aging upon saccadic suppression were not systematically investigated before. So far, only one other study investigated developmental effects upon saccadic suppression. Bruno et al. (2006) compared luminance and chromatic contrast sensitivity during fixation and saccades in groups of three children, 10 adolescents (12–14 years), three older adolescents (15–18), and 10 young adults (21–31 years). The authors used large horizontally oriented Gabor stimuli (35° wide and 24.5° high) of low spatial frequency (0.15°) as detection targets that were flashed during fixation and about 20 ms after the onset of rightward saccades to targets appearing at 16° eccentricity. The Gabor stimuli used for detection were either modulated in luminance or chromaticity. The authors found that compared to fixation, contrast sensitivity was significantly reduced during saccades only for luminance. For adolescents saccadic suppression of luminance was stronger than for young adults (90%–95% for adolescents vs. 70%–90% for adults).

Overall, Bruno et al. (2006) found stronger suppression than we did. This may be partially caused by the lower spatial frequency of their luminance Gabor target compared to our Gaussian line stimulus, or by the more peripheral location of their Gabor target. They did observe significant differences in suppression between their group of adolescents (93.9%) and the young adults (81.9%). We did not observe a significant difference between our group of adolescents (≤17 years) and the young adults. In our study, the strength of saccadic suppression was very similar for adolescents (65.5%), young adults (64%), and senior adults (70.2%). However, there was a post-hoc trend for the observers up to 14 years old to show slightly higher suppression (see Figure 7), whereas the adolescents between 15 to 17 years old exhibit a larger variability in their suppression effects, similar to what Bruno et al. (2006) observed. Because we could recruit only two adolescents between 12 to 14 years of age, for which Bruno et al. (2006) reported their significant effects, our data do not disagree with the earlier findings of there being stronger suppression at the earliest ages.

### Saccadic suppression of color

While previous studies reported a selective saccadic suppression for low frequency luminance stimuli only, with a sparing of color and high spatial frequency stimuli (Burr et al., 1994; Diamond et al., 2000; Bruno et al., 2006), we found a consistent saccadic suppression also for color, albeit not as strong as luminance suppression (Braun et al., 2017; see also the conference abstract of Rolfs & Castet, 2014). We think that these differences in results with respect to chromatic stimuli might be based on the specific experimental conditions used, that is, flash duration, location and size of detection stimuli, stimulus background and the light conditions in the experimental room (see Table 2 in Braun et al., 2017). Bruno et al. (2006) used large brown or red-green gratings as detection stimuli on a yellow background, similar to earlier studies of the group (Burr et al., 1994; Diamond et al., 2000). They used individual flicker photometry to estimate isoluminance for the chromatic stimuli and reported no change of contrast sensitivity during saccades for chromatic stimuli.

Direct comparisons of saccadic suppression effects between studies are limited by the fact that not only saccade tasks and test conditions but also the detection stimuli differ to large extents (see table 2 of Braun et al., 2017). For example, saccadic suppression was measured during saccades to a single target appearing always at the same location (Diamond et al., 2000; Knöll et al., 2011), during saccades alternating between two constant locations in a Ganzfeld (Volkmann et al., 1978), and during saccades in a free viewing condition when watching a movie (Dorr & Bex, 2013). As detection stimuli light flashes, single line targets, Gaussian blobs, Gabor patches or gratings up to a size of 35 × 25 deg were presented for durations of 8 ms to 26 ms.

In the study of Bruno et al. (2006), the chromatic Gabor stimulus was presented five times longer than their luminance stimulus. This was probably necessary to deal with the lower contrast sensitivity of color for their more peripheral targets. In our study, we used the same duration of 8 ms for both, luminance and color flashes and used a slightly more greenish background that allowed for much larger chromatic contrast variations. On average we found a chromatic suppression of 36%. Interestingly, the data in Figure 3 of Bruno et al. (2006) do show a trend for chromatic suppression of about 22% for the group of adolescents and of 16% for young adults. The smaller amount of chromatic suppression may result from their five times longer flash duration of the chromatic stimulus or from an overall larger eccentricity of their Gabor target. However, it has to be kept in mind that the variability of chromatic suppression seems larger in general. In their group of nine adults, five participants showed suppression for color between 17% to 50%, two had no suppression, and two showed an enhancement of 50% to 70%. For all adolescents but one, saccadic suppression of color was present and reached about 24 % on average. Thus the differences between the results of Bruno et al. (2006) and our present results might not be that large after all.

Our results presented here for saccadic suppression of color do agree perfectly with our earlier experiments (Braun et al., 2017), where we also carefully dealt with issues of individual isoluminance. Therefore, we think that saccadic suppression is not an exclusive mechanism for luminance (Ross, Burr, & Morrone, 1996; Bruno et al., 2006; Knöll et al., 2011) but that suppression is present also for low frequency chromatic stimuli, albeit at lower magnitude and larger variability. There are other potential reasons why we would find saccadic suppression for color, and why color suppression is smaller than for luminance. One might argue that the location of our saccade targets was not predictable and that the saccade task might thereby be more demanding. This could lead to dual-task costs, which in our case would show up as saccadic suppression for color. In this case, only the added suppression for luminance would be due to real suppression, according to our findings this would reduce contrast sensitivity for luminance by only 30%. Along similar lines, it could be argued that saccadic suppression might be due to retinal shearing of photoreceptors (Castet, Jeanjean, & Masson, 2001), whereas active suppression is then only added for the luminance stimuli. Both of these arguments would predict that there should not be any condition under which there is no saccadic suppression at all. However, in our earlier experiments (Braun, Schütz, & Gegenfurtner, 2017), and in agreement with the literature (Burr, Morrone, & Ross, 1994; Volkmann et al., 1978), we found that there is minimal suppression for high spatial frequency targets, using identical experimental conditions as in this experiment.

### Relationship to electrophysiology

Color and luminance information are often associated with different processing streams, that is, the magnocellular and parvocellular pathways starting in the LGN (DeYoe & Van Essen, 1988; Maunsell, Nealey & DePriest, 1990; Schiller & Logothetis, 1990; see Merigan & Maunsell, 1993; Callaway, 2005; for reviews). Although magnocellular neurons of the lateral geniculate nucleus are tuned to luminance stimuli of low spatial frequencies, parvocellular neurons are tuned to high spatial frequencies and to color. This has led to the hypothesis of a specific suppression of the magnocellular pathway (Burr, Morrone, & Ross, 1994; Ross et al., 2001), but neurophysiological studies provided mixed results. In the lateral geniculate nucleus, magnocellular and parvocellular neurons show a perisaccadic decrease in response amplitudes (Reppas, Usrey, & Reid, 2002; Saul, 2010). Electrophysiological recordings of neurons in area V4 by Han, Xian and Moore (2009) revealed very heterogeneous effects of saccade preparation on neuronal color contrast sensitivity. When these authors compared the dynamics of luminance and chromatic contrast sensitivity of V4 neurons in monkey before the onset of saccades, they observed a general decrease in luminance sensitivity 50 ms before saccade onset while for color the decrease was smaller and more variable and some neurons showed no or only little change of their neuronal response properties. In the frontal eye field presaccadic suppression of visual contrast sensitivity was present for both color and luminance stimuli in visual cells but not in visuomotor cells (Krock & Moore, 2016). In a human functional magnetic resonance imaging (fMRI) study Kleiser, Seitz and Krekelberg (2004) reported comparable saccadic suppression in V1 BOLD responses and a selective suppression for luminance in area MT and V4 when participants had the task to make saccades to luminance and isoluminant color targets. Taken together, the processing of color information involves more areas along the ventral stream and seems to be more distributed compared to the processing of motion information (Zeki, 1983; Chaparro et al., 1993; Gegenfurtner & Kiper, 2003; Shapley & Hawken, 2011). With respect to saccadic suppression, modulations of neuronal responses to color stimuli of V4 neurons seem to be more variable and our result of smaller and variable suppression effects for color stimuli is therefore in line with results of the neurophysiological studies as summarized in a recent review of Krock and Moore (2014).

### Vision and age

Luminance and chromatic detection sensitivity depend on intact functions of cone photoreceptors, retina, the visual pathways and cortical areas. Across the lifespan a u-shaped function was found for chromatic sensitivity, which improves in adolescence until a maximum is reached between 20–30 years followed by a gradual decrease. Children at the age around five to six years have a relatively mature retina and neural connections between the well-developed visual cortex and related areas (Graven, 2004). However, humans reach relatively late at about 20 years of age their best chromatic detection sensitivity and after about 40 years of age chromatic detection sensitivity starts to decline constantly around 1% per year during healthy aging (Knoblauch, Vital-Durand, & Barbur, 2001; Paramei & Oakley, 2014), and the decrease seems to be mainly caused by noise (Schefrin, Shinomori & Werner, 1995) and a loss of retinal ganglion axons (Jonas, Müller-Bergh, Schlötzer-Schrehardt, & Neumann, 1990). In the present study we also found a significant decrease of detection contrast sensitivity for luminance and color and an increase of saccadic latencies for our senior participants, however, our results indicate that mechanisms for saccadic suppression seem to be very stable with respect to healthy aging. This finding is quite surprising taking the wealth of psychophysical evidence of decline of visual sensitivity during normal aging and the given complexity of the neural network behind saccadic suppression (Stanford & Pollack, 1984; Kline & Schieber, 1985; Spear, 1993; Wist, Schrauf, & Ehrenstein, 2000; Sekuler & Sekuler, 2000; Norman, Ross, Hawken, & Long, 2003; Owsley, 2011; Rey-Mermet & Gade, 2018). A lot of work has been done to investigate the location and mechanism of the degradation affecting specific visual functions during aging in man (Owsley, Sekuler, & Siemens, 1983; Elliot & Werner, 2010) and monkey (Schmolesky, Wang, Pu, 2000; Leventhal, Wang, Pu, Zhou, & Ma, 2003; Yu, Wang, Li, Zhou, & Leventhal, 2006), which are not caused by the deterioration of the optical quality of the eyes (Weale, 1961; Elliott, Whitaker & MacVeigh, 1990; Weale, 1992). One example is the aging impairments found for the sensitivity of visual motion; i.e. reduced abilities to detect or identify the direction of movement or to differentiate speeds (Trick & Silverman, 1991; Andersen & Atchley, 1995; Atchley & Andersen, 1998; Norman, Ross, Hawkes, & Long, 2003; Raghuram, Lakshminarayanan, & Khanna, 2005; Snowden & Kavanagh, 2006; Bennett, Sekuler, & Sekuler, 2007). Betts, Taylor, Sekuler, and Bennett (2005) used a motion discrimination task previously developed by Tadin, Lappin, Gilroy and Blake (2003) to test age-related changes on center-surround interactions. For large high-contrast patterns they found that older participants needed shorter durations of the motion stimulus than younger participants to perceive the movement direction. Their finding indicates that age reduces the efficacy of cortical inhibitory mechanisms, which exert strong spatial suppression for large stimuli at high contrast in young observers but which result in the older adult with decreased cortical inhibition in a better performance in this specific task. Age effects are also well documented for saccadic latency, accuracy, velocity, and errors (Munoz et al., 1998; Klein & Foerster, 2001; Irving et al., 2006; Peltsch et al., 2011), and they are also present in our data. Although we had expected a decrease of saccadic suppression with age for senior adults together with an increase in mean saccadic latencies and decreased contrast sensitivity, saccadic suppression slightly increased for luminance or stayed stable for color. Therefore we conclude that the mechanisms of saccadic suppression are remarkable stable with respect to healthy aging.

## Conclusion

We studied saccadic suppression of visual contrast sensitivity for briefly flashed low-spatial frequency luminance and isoluminant color stimuli in a large group of participants over a large age range from eight to 78 years. Saccadic suppression was clearly stimulus specific, that is, it was about two times stronger and less variable for luminance flashes compared to isoluminant chromatic flashes. We found a high stability of saccadic suppression with respect to healthy ageing and a strong intersubject variability concerning the strength of luminance and in particular color suppression during saccades.

## Footnotes

1 Correlations in linear contrast sensitivity were only slightly lower in value by 0.05 in both cases and also highly significant.
